# Metabolic, adipokine (irisin, spexin, visfatin), and ER stress (GRP78) responses in multiparous Brown Swiss cows: a multivariate approach across physiological stages and body condition scores

**DOI:** 10.5194/aab-69-517-2026

**Published:** 2026-09-17

**Authors:** Gökşad Cemil Kotan, Şeyma Aydemir, Bülent Bayraktar

**Affiliations:** 1 Alaca Avni Çelik Vocational School, Department of Veterinary Medicine, Hitit University, Çorum, 19000, Türkiye; 2 Faculty of Health Sciences, Department of Physiotherapy and Rehabilitation, Bayburt University, Bayburt, 69000, Türkiye

## Abstract

This study investigated the responses of novel adipokines (irisin, spexin, visfatin), an endoplasmic reticulum (ER) stress marker (GRP78), and traditional metabolic parameters (non-esterified fatty acids (NEFAs), beta-hydroxybutyrate (BHBA), urea, albumin, globulin, total cholesterol) in 360 healthy Brown Swiss dairy cattle. The animals were categorized into 18 subgroups based on pregnancy stages (early, mid, dry), lactation stages (early, mid, late), and body condition scores (BCSs). Serum analyses were performed using ELISA and spectrophotometric methods. Interactions between physiological stages and BCS were highly significant for all measured parameters ( p<0.001 ). Results showed that mean serum levels of spexin (ranging from 14.02 to 36.04 pg mL^−1^) and irisin increased as pregnancy and lactation progressed. Visfatin levels exhibited a significant increase specifically during the gestation period, peaking during the dry period (24.09 ng mL^−1^), and maintained a significant increasing trend as lactation progressed toward the late stage (Tables 2, 4). Conversely, GRP78 (peaking at 0.46 ng mL^−1^), NEFA (peaking at 0.52 mmol L^−1^), and urea levels peaked during the dry period and early lactation, subsequently decreasing as metabolic stability was achieved. Notably, during the dry period, stress markers rose despite declining albumin and globulin levels ( p<0.05 ). These data indicate that the dry period and early lactation are the most critical phases for metabolic and cellular stress, characterized by lower spexin and elevated GRP78 and NEFA concentrations. The later rise in irisin and visfatin, while BHBA remained within physiological limits, confirms successful metabolic adaptation ( p<0.001 ). In conclusion, the integrated evaluation of these novel biomarkers is anticipated to provide significant contributions to protective strategies and metabolic management in dairy cattle.

## Key points

- Spexin, visfatin, and GRP78 are potential biomarkers for bovine metabolic status.
- Physiological stages and BCS significantly modulate adipokines and GRP78.
- Peak NEFA in early lactation coincides with low irisin and spexin levels.
- High BCS exacerbates endoplasmic reticulum stress during early lactation.
- Serum spexin, irisin, and visfatin levels increase as lactation progresses, whereas GRP78 peaks during early lactation and the dry period, highlighting these as key physiological stress points

## 1 Introduction

Pregnancy and lactation periods in dairy cattle represent a critical transition phase where physiological adaptation and metabolic capacity are utilized to their maximum extent, leading to an increased risk of negative energy balance (NEB) (Bayraktar et al., 2020; Losacco et al., 2025). The onset of early lactation, in particular, is characterized by the NEB phenomenon, resulting from the inability to meet the high energy demand required for milk synthesis through limited dry matter intake (Mekuriaw, 2023). To compensate for this energy deficit, intense lipolysis is initiated; however, this process elevates serum non-esterified fatty acid (NEFA) and beta-hydroxybutyrate (BHBA) levels, thereby increasing the oxidative load on the liver and suppressing metabolism. This triggers metabolic stress and clinical or subclinical complications of NEB, such as ketosis, fatty liver syndrome, abomasal displacement, metritis, and mastitis (Ji et al., 2023). Consequently, immunosuppression leads to infectious diseases, while weakened reproductive functions result in fertility losses (Potiris et al., 2025). The resulting yield losses and high treatment costs, combined with increased involuntary culling rates, threaten the sustainability and profitability of dairy enterprises (Kang et al., 2025).

Adipose tissue is a metabolically active endocrine organ that regulates energy homeostasis and thermoregulation through adipokines (Bayraktar, 2020). Spexin (SPX, neuropeptide Q) is a novel adipokine with pleiotropic functions in regulating insulin resistance, glucose levels, blood pressure (Liu et al., 2022), lipid and energy metabolism (Sun et al., 2023), body weight control (Jeong et al., 2022), and metabolic stress responses (Kolodziejski et al., 2018; Türkel et al., 2022). Spexin plays a role in preventing hepatic lipid accumulation by stimulating lipolysis and inhibiting lipogenesis (Pruszynska-Oszmalek et al., 2020). Irisin is a proteolytic product of the fibronectin type III domain-containing protein 5 (FNDC5); it is an adipo-myokine that increases energy expenditure and thermogenesis by inducing the browning of white adipose tissue, often stimulated by exercise (Boström et al., 2012; Öztüfek et al., 2019). It serves as an indicator molecule in the regulation of body mass index and energy metabolism (Bayraktar and Tekce, 2020). Visfatin (NAMPT) is an adipokine with insulinomimetic and pro-inflammatory effects, primarily expressed in visceral adipose tissue. It exists in intracellular (iNAMPT) and extracellular (eNAMPT) forms and plays a role in glucose homeostasis and metabolic response regulation (Ratajczak-Pawłowska et al., 2025; Ormazabal et al., 2025). While iNAMPT coordinates cellular survival and nicotinamide adenine dinucleotide (NAD)-dependent activities, eNAMPT is secreted from various tissues, particularly adipose tissue, exerting endocrine and paracrine effects (Shi and Gu, 2025). Visfatin acts as an energy balance regulator by increasing glucose utilization through NAD biosynthesis and insulin-like effects (Komarnicki et al., 2025).

Endoplasmic reticulum (ER) stress occurs when protein folding is disrupted within the ER, directly impacting metabolic health (Martinotti and Ranzato, 2025). GRP78 (Glucose-Regulated Protein 78), also known as BiP or HSPA5, is a 78 kDa member of the HSP70 family (Lin et al., 2025). Beyond its role as an ER stress sensor, GRP78 is involved in protein folding, the unfolded protein response (UPR), regulation of protein synthesis and degradation, and calcium homeostasis (Vietri et al., 2025; Byun et al., 2025). NEFA is a primary indicator of lipolysis; elevated blood levels confirm that the animal has begun mobilizing fat stores due to NEB, serving as an early prognostic marker for ketosis and fatty liver (Chen et al., 2025). BHBA, one of the three main ketone bodies produced by the liver during periods of low carbohydrate intake, fasting, or prolonged exertion, indicates the liver's fatty acid oxidation capacity (Li et al., 2025; Gregor et al., 2025). Excessive BHBA elevation reveals the severity of the energy deficit and the risk of subclinical or clinical ketosis while signaling an increased metabolic load on the liver and the risk of hepatic lipidosis (Sosa-Higareda and Beaufrère, 2025). Parameters such as urea (reflecting protein metabolism and dietary energy balance; Slivinska et al., 2025), albumin (indicating hepatic synthesis capacity and nutritional status; Cameron et al., 2025), globulin (as a marker of immune response and inflammation; Tani et al., 2025), and total cholesterol (reflecting liver health and lipid transport; Wang et al., 2025) are critical for early diagnosis and constitute the general metabolic profile of the animal (Ünal et al., 2026). During the life cycle of cattle, especially in early lactation, there is a close relationship between health status, fertility, production performance, and body energy reserves (Xu et al., 2019). Body condition score (BCS) is one of the most common field methods for evaluating subcutaneous fat deposits and metabolic health in dairy cattle (Ferguson et al., 1994; Bayraktar and Genç, 2021; Sun et al., 2025). BCS can be determined through inspection, palpation, ultrasonography, or needle measurements. Among these, palpation and inspection are subjective techniques based on the appearance of the rump, tailhead, lumbar vertebrae, and ribs (Edmonson et al., 1989). The BCS system numerically classifies cattle fatness on a scale from 1 (emaciated) to 5 (obese) (Butler and Smith, 1989). Furthermore, adipose-derived adipokines and cellular stress responses also play pivotal roles in metabolic adaptation mechanisms (Turk et al., 2008; Xu et al., 2019; Goselink et al., 2020). In this context, novel adipokines such as spexin, irisin, and visfatin are thought to regulate glucose and lipid metabolism, influencing insulin resistance and energy homeostasis. In particular, the inhibitory effects of spexin on energy intake and fat metabolism (Harooni and Radmehr, 2025) suggest its potential as a biomarker for metabolic stress during lactation and pregnancy (Elsherbiny et al., 2025; Chen et al., 2025). Although numerous studies exist regarding the metabolic profile of cattle, comprehensive research examining the effects of different condition scores on novel markers such as spexin, irisin, visfatin, and GRP78 throughout the pregnancy and lactation stages in Brown Swiss cattle is quite limited. Therefore, this study aims to provide a new perspective on the physiological adaptation processes of Brown Swiss cattle by evaluating adipokine responses (spexin, irisin, and visfatin) and ER stress levels (GRP78) in conjunction with metabolic parameters (NEFA, BHBA, urea, albumin, globulin, total cholesterol) throughout the lactation and pregnancy cycles.

## 2 Materials and methods

### 2.1 Animals

The animal material of the study consisted of 360 healthy multiparous Brown Swiss dairy cows, raised under intensive conditions in a large-scale commercial dairy enterprise. The enterprise is located in Çorum, Türkiye (40°33^′^ N, 34°57^′^ E) and maintains detailed animal tracking records. Ethical approval for the study was obtained from the Local Ethics Committee of the Veterinary Control Central Research Institute (date: 13 May 2022; decision no.: 2022/13). All experimental procedures were conducted in strict accordance with ethical principles and international guidelines, ensuring the protection of animal welfare and rights.

#### 2.1.1 Experimental design

The study was conducted on a total of 360 healthy multiparous Brown Swiss cows, which were systematically categorized into 18 distinct subgroups ( n=20 per group) based on a multivariate experimental design that integrated six primary physiological stages. These stages comprised three pregnancy periods (early: 1–90 d; mid: 91–210 d; dry period: the final 60 d before expected calving) and three lactation periods (early: 1–100 d postpartum; mid: 101–200 d postpartum; late: from day 201 until drying off). Each physiological stage was cross-referenced with three specific body condition score (BCS) categories (low, moderate, and high) to evaluate metabolic, adipokine, and ER stress responses. To minimize intra-observer variation, BCS assessments were performed by a single expert using a five-point scoring system through visual inspection and palpation of the lumbosacral and pelvic regions. Detailed chemical composition and nutritional values of the diets provided during these experimental stages are presented separately in Table 1. The BCS cut-off values were dynamically adjusted across gestation and lactation stages to accurately reflect the physiological weight shifts, body reserve mobilization, and changing optimal condition targets expected throughout the production cycle of dairy cattle.

**Table 1:** Nutrient composition and chemical analysis of the ration (g kg^−1^).

Feed ingredients	Dietary feed	Ration
(kg)	(lactation)	(pregnancy/dry)
Corn silage (30 %–35 % dry matter, DM)	22	–
Oat hay	5	–
Barley/corn meal	2.00	–
Oat silage	–	10
Wheat straw	–	8
Dairy feed (19 CP (crude protein), 2700 kcal kg^−1^ metabolizable energy (ME))	8.00	1.5
Dairy feed (21 CP, 2750 kcal kg^−1^ ME)	6.5	–
Soybean meal (44 % HP)	1.5	–
Sunflower meal (32 % HP)	–	1.5
Premix powder	1.00	1.00
Total (kg)	38.80	21.10
DM (kg)	21.50	12.80
Crude protein (CP) (%)	17.5–18.0	12.5–13.0
Energy (ME) (kcal kg^−1^)	2720	2250

#### 2.1.2 Feed

The feed ingredients were provided by a private company, and the chemical compositions of the diets were analyzed. The dry matter, crude ash, crude protein, and crude fat contents were determined using the methods reported by AOAC International (2016). The crude fiber content was analyzed following the procedure described by Crampton and Maynard (1938). Neutral detergent fiber (NDF) and acid detergent fiber (ADF) contents were measured according to the methods of Van Soest et al. (1991) and Goering and Van Soest (1970), respectively. The comprehensive nutrient composition and chemical analysis of the diets for the pregnant/dry and lactation periods are detailed in Table 1.

#### 2.1.3 Collection of serum samples

Blood samples were collected from the jugular vein into 10 mL vacuum tubes containing a clot activator (VACUETTE^®^ TUBE 9 mL Z serum clot activator, Greiner Bio-One, Austria) to determine the levels of spexin, irisin, visfatin, GRP78, NEFA, BHBA, urea, albumin, globulin, and total cholesterol. Following collection, the blood samples were centrifuged at + 4 °C at 3000 rpm for 10 min to separate the serum (NF 1200R, Nüve, Ankara, Türkiye). The resulting serum samples were transferred into Eppendorf tubes and stored at - 80 °C until laboratory analysis (Bayraktar and Genç, 2021).

#### 2.1.4 Biochemical analysis

Serum concentrations of NEFA, BHBA, urea, albumin, globulin, and total cholesterol were measured using a Cobas 8000 modular analyzer series (Roche Diagnostics, Mannheim, Germany), employing a closed photometric system in accordance with the manufacturer's protocols (Kaneko et al., 2008).

#### 2.1.5 Measurement of serum spexin, irisin, visfatin, and GRP78 levels

Serum spexin, irisin, visfatin, and GRP78 concentrations were measured using commercial bovine-specific ELISA kits (YL Biont, Shanghai, China) and evaluated with a microplate reader (Mindray MR-96A, China/R&D Systems, USA). The minimum detectable concentration used to measure the spexin (C12orf39) level in blood serum obtained from the research was 0.12 pg mL^−1^. An ELISA kit type specific to bovine spexin (YL Biont, catalog no. YLA0409BO, China) with an assay range of 0.2–60 pg mL^−1^, an intra-assay coefficient of <8.0 %, and an inter-assay coefficient of <10.0 % was utilized in accordance with the manufacturer's protocol. The results were evaluated by reading absorption values at 450 nm in accordance with the procedure reported in the kit (Spexin, 2025).

The minimum detectable concentration used to measure the Irisin level in blood serum obtained from the research was 2.63 ng mL^−1^. An ELISA kit type specific to bovine irisin (YL Biont, catalog no. YLA0410BO, China) with an assay range of 5–1000 ng mL^−1^, an intra-assay coefficient of <8.0 %, and an inter-assay coefficient of <10.0 % was utilized in accordance with the manufacturer's protocol. The results were evaluated by reading absorption values at 450 nm in accordance with the procedure reported in the kit (Irisin, 2025).

The minimum detectable concentration used to measure the Visfatin level in blood serum obtained from the research was 1.05 ng mL^−1^. An ELISA kit type specific to bovine visfatin (YL Biont, catalog no. YLA0411BO, China) with an assay range of 2–160 ng mL^−1^, an intra-assay coefficient of <8.0 %, and an inter-assay coefficient of <10.0 % was utilized in accordance with the manufacturer's protocol. The results were evaluated by reading absorption values at 450 nm in accordance with the procedure reported in the kit (Visfatin, 2025).

The minimum detectable concentration used to measure the GRP78 level in blood serum obtained from the research was 0.037 ng mL^−1^. An ELISA kit type specific to bovine glucose-regulated protein 78 (GRP78) (YL Biont, catalog no. YLA0413BO, China) with an assay range of 0.1–38 ng mL^−1^, an intra-assay coefficient of <8.0 %, and an inter-assay coefficient of <10.0 % was utilized in accordance with the manufacturer's protocol. The results were evaluated by reading absorption values at 450 nm in accordance with the procedure reported in the kit (GRP78, 2025).

#### 2.1.6 Statistical analysis

Statistical analysis of the data was performed using the SPSS 22.0 software package (IBM Corp., Armonk, NY, USA). The normality of the data distribution was assessed using the Shapiro–Wilk test. Descriptive statistics are expressed as mean and standard error of the mean (SEM). To determine the effects of physiological stages, body condition scores (BCSs), and their interactions, a two-way factorial ANOVA using the general linear model (GLM) procedure was employed. The mathematical model was defined as

$$Y_{ijk}=\mu +P_{i}+B_{j}+(P\times B{)}_{ij}+e_{ijk},$$

where

-
$Y_{ijk}$ is the observation value,
-
μ is the overall mean,
-
$P_{i}$ is the effect of the period ( i=1 , 2, 3 for gestation stages or lactation stages),
-
$B_{j}$ is the effect of the BCS category ( j=1 , 2, 3 for low, moderate, and high),
-
$(P\times B{)}_{ij}$ is the interaction effect between the period and BCS,
- eijk is the random experimental error.

Duncan's multiple range test was used as a post hoc analysis to identify specific differences between group means. Statistical significance was set at p<0.05 .

## 3 Results

### Spexin, irisin, visfatin, GRP78, and selected blood parameters

Serum levels of spexin, irisin, visfatin, and GRP78 according to lactation stages and BCS groups are presented in Table 2. The highest mean serum spexin level was determined in the late-lactation BCS > 3.75 group (36.04 pg mL^−1^), whereas the lowest mean level was detected in the early-lactation BCS 2.5–3.0 group (18.24 pg mL^−1^; Table 2).. The lowest mean serum irisin level was identified in the early-lactation BCS < 2.5 group (0.95 ng mL^−1^), while the highest level was measured in the late-lactation BCS > 3.75 group (2.07 ng mL^−1^). In contrast, mean serum visfatin levels were lowest in the early-lactation group and highest in the late-lactation group, reaching their peak as metabolic stability was achieved ( p<0.001 ). Furthermore, it was determined that serum GRP78 levels were significantly influenced by both lactation periods and BCS ( p<0.001 ). Mean serum GRP78 levels, an indicator of endoplasmic reticulum stress, reached their peak in the BCS < 2.5 group (low condition score) during early lactation (0.23 ng mL^−1^) and were at their minimum in the BCS > 3.75 group (high condition score) during late lactation (0.12 ng mL^−1^) (Table 2).

**Table 2:** Mean serum levels of spexin, irisin, visfatin, and GRP78 in Brown Swiss cattle across different lactation stages and BCS groups (Mean ± SEM).

Parameters	Early lactation	Early lactation	Early lactation	Mid-lactation	Mid-lactation	Mid-lactation	Late lactation	Late lactation	Late lactation	SEM	p value
	( <2.5 )	(2.5–3.0)	( >3 )	( <2.75 )	(2.5–3.25)	( >3.25 )	( <3.25 )	(3.25–3.75)	( >3.75 )		
Spexin (pg mL^−1^)	23.18^f^ ± 0.31	21.05^g^ ± 0.31	18.24^h^ ± 0.31	25.13^e^ ± 0.31	27.24^d^ ± 0.31	29.87^c^ ± 0.31	31.25^c^ ± 0.31	34.02^b^ ± 0.31	36.04^a^ ± 0.31	0.31	<0.001
Irisin (ng mL^−1^)	1.14^g^ ± 0.02	1.11^g^ ± 0.02	1.08^g^ ± 0.02	1.25^f^ ± 0.02	1.34^e^ ± 0.02	1.48^d^ ± 0.02	1.55^d^ ± 0.02	1.78^b^ ± 0.02	1.95^a^ ± 0.02	0.02	<0.001
Visfatin (ng mL^−1^)	14.28^f^ ± 0.03	12.14^g^ ± 0.03	10.05^h^ ± 0.03	16.55^e^ ± 0.03	18.24^d^ ± 0.03	20.15^c^ ± 0.03	22.34^b^ ± 0.03	24.55^b^ ± 0.03	26.87^a^ ± 0.03	0.03	<0.001
GRP78 (ng mL^−1^)	0.23^a^ ± 0.02	0.20^b^ ± 0.02	0.18^c^ ± 0.02	0.16^d^ ± 0.02	0.15^d,e^ ± 0.02	0.14^*e* *f*^ ± 0.02	0.14^*e* *f*^ ± 0.02	0.13^f^ ± 0.02	0.12^g^ ± 0.02	0.02	<0.001

^*^ EL: early lactation; ML: mid-lactation; LL: late lactation; BCS: body condition score; SEM: standard error of the mean. GRP78: 78 kDa glucose-regulated protein (ER stress marker). Data were analyzed independently for each lactation stage and BCS group. Statistical significance was set at p<0.05 . ^a–h^ Means within the same row with different superscript letters differ significantly ( p<0.05 ).

The mean serum levels of metabolic response parameters (NEFA, BHBA, urea, albumin, globulin, and total cholesterol) across lactation stages and BCS groups in Brown Swiss cattle are presented in Table 3. The highest mean NEFA levels were determined in the BCS > 3 group (0.41 mmol L^−1^) during the early lactation period, when negative energy balance (NEB) was most pronounced ( p<0.001 ). While serum NEFA levels in the early-lactation groups remained below the physiological threshold of 0.40 mmol L^−1^ (Table 3), a higher mean level was observed during the dry period (0.52 mmol L^−1^; Table 5), reflecting the increased lipolysis during the transition to the next lactation cycle. Conversely, the lowest NEFA levels were determined in the mid-lactation BCS < 2.75 group (0.21 mmol L^−1^, p<0.001 ). Regarding mean serum BHBA levels during lactation, the values ranged from 0.21 mmol L^−1^ in the late-lactation groups to 0.48 mmol L^−1^ in the early-lactation high-condition group ( p<0.001 ; Table 3). In terms of protein metabolism, serum urea levels were lowest in the BCS < 3.25 group (21.36 mg dL^−1^) during late lactation, while the highest levels were found in the BCS 2.5–3.0 group (26.45 mg dL^−1^) during early lactation ( p<0.001 ). The lowest serum albumin and globulin levels were detected in the BCS < 2.5 group during early lactation (32.72 and 30.93 g L^−1^, respectively), while the highest levels were observed in the late-lactation group with BCS > 3.75 (38.08 and 36.36 g L^−1^, respectively) ( p<0.05 ). The lowest mean serum total cholesterol levels were found in the BCS < 2.5 group (102.2 mg dL^−1^) during early lactation, while the highest level was found in the BCS > 3.75 group (155.2 mg dL^−1^) during late lactation ( p<0.001 ; Table 3).

**Table 3:** Changes in mean serum NEFA, BHBA, urea, albumin, and total cholesterol levels in Brown Swiss cows across different lactation stages and BCS groups (mean ± SEM).

Parameters	Early lactation			Mid-lactation			Late lactation			SEM	p value
	BCS groups			BCS groups			BCS groups				
	<2.5	2.5–3.0	>3	<2.75	2.75–3.25	>3.25	<3.25	3.25–3.75	>3.75		
NEFA (mmol L^−1^)	0.34^c^	0.38^b^	0.41^a^	0.21^e^	0.34^c^	0.37^b^	0.23^d^	0.25^d^	0.30^c^	0.01	<.001
BHBA (mmol L^−1^)	0.38^b^	0.42^a,b^	0.48^a^	0.24^d,e^	0.31^c,d^	0.35^b,c^	0.21^e^	0.22^e^	0.25^d,e^	0.02	< 0.001
Urea (mg dL^−1^)	22.15^a^	26.45^a^	25.63^a^	24.15^a^	24.75^a^	25.26^a^	21.36^b^	22.12^b^	23.42^b^	0.16	<.001
Albumin (g L^−1^)	32.72^d^	34.77^b,c^	35.83^b^	34.15^c^	35.64^b^	36.32^a,b^	35.12^b^	36.32^a,b^	38.08^a^	0.28	0.012
Globulin (g L^−1^)	30.93^c^	32.04^c^	34.79^b^	32.52^b^	34.88^a,b^	35.62^a^	34.83^a,b^	35.69^a^	36.36^a^	0.31	0.035
Total cholesterol (mg dL^−1^)	102.2^e^	118.9^d^	122.7^d^	110.0^e^	121.8^d^	135.2^c^	153.2^a^	153.7^a^	155.2^a^	0.24	<.001

^*^ NEFA: non-esterified fatty acid; BHBA: beta-hydroxybutyric acid; EL: early lactation; ML: mid-lactation; LL: late lactation; BCS: body condition score. Statistical analysis: data were analyzed independently for each physiological period and BCS group. Statistical significance was set at p<0.05 . Different superscript letters (a–e) within the same row indicate statistically significant differences between groups ( p<0.05 ).

The mean serum spexin, irisin, visfatin, and GRP78 levels in Brown Swiss cattle with different gestation stages and BCS groups in the study group are presented in Table 4. The lowest mean spexin level was found in the BCS > 3 group during early pregnancy (14.02 pg mL^−1^), while the highest level was detected in the BCS < 3.25 group during the dry period of pregnancy (36.04 pg mL^−1^). Regarding irisin, a hormone associated with energy metabolism, the lowest levels were identified in the early-gestation BCS < 2.5 group (2.01 ng mL^−1^), while the highest levels were determined in the dry-period BCS > 3.75 group (2.66 ng mL^−1^) ( p<0.001 ). The mean serum visfatin level for different periods of pregnancy was lowest in the mid-gestation BCS 2.75–3.25 group (21.24 ng mL^−1^), while the highest level was detected in the dry-period BCS > 3.75 group (24.09 ng mL^−1^) ( p<0.001 ). The mean serum GRP78 levels, a cellular stress marker, were lowest in the early-gestation BCS > 3.75 group (0.18 ng mL^−1^), while the highest level was observed in the dry period of gestation in the BCS < 3.25 group (0.46 ng mL^−1^) ( p<0.001 ; Table 4).

**Table 4:** Mean serum levels of spexin, irisin, visfatin, and GRP78 in Brown Swiss cattle across different gestation stages and BCS groups (mean ± SEM).

Parameters	Early pregnancy	Early pregnancy	Early pregnancy	Mid-pregnancy	Mid-pregnancy	Mid-pregnancy	Dry period	Dry period	Dry period	SEM	p value
	( <2.5 )	(2.5–3.0)	( >3 )	( <2.75 )	(2.75–3.25)	( >3.25 )	( <3.25 )	(3.25–3.75)	( >3.75 )		
Spexin (pg mL^−1^)	23.13^f^ ± 0.31	18.05^h^ ± 0.31	14.02^i^ ± 0.31	29.09^c^ ± 0.31	26.05^e^ ± 0.31	22.03^f^ ± 0.31	36.04^a^ ± 0.31	33.80^a^ ± 0.31	30.04^b^ ± 0.31	0.31	<.001
Irisin (ng mL^−1^)	2.01^d^ ± 0.01	2.05^d^ ± 0.01	2.08^d^ ± 0.01	2.18^c^ ± 0.01	2.27^*b* *c*^ ± 0.01	2.34^b^ ± 0.01	2.42^b^ ± 0.01	2.47^b^ ± 0.01	2.66^a^ ± 0.01	0.01	<.001
Visfatin (ng mL^−1^)	21.23^c^ ± 0.25	21.30^c^ ± 0.25	21.87^c^ ± 0.25	22.15^b^ ± 0.25	21.24^c^ ± 0.25	22.15^b^ ± 0.25	23.61^a^ ± 0.25	23.63^a^ ± 0.25	24.09^a^ ± 0.25	0.25	<.001
GRP78 (ng mL^−1^)	0.24^c^ ± 0.02	0.21^c^ ± 0.02	0.18^d^ ± 0.02	0.29^b^ ± 0.02	0.26^b^ ± 0.02	0.22^c^ ± 0.02	0.46^a^ ± 0.02	0.41^a^ ± 0.02	0.38^a^ ± 0.02	0.02	<.001

^*^ EGP: early gestation period; MGP: mid-gestation period; dry: late gestation/dry period; BCS: body condition score; SEM: standard error of the mean. GRP78: 78 kDa glucose-regulated protein (endoplasmic reticulum stress marker). Data were analyzed independently for each gestation stage and BCS group. Statistical significance was set at p<0.05 . ^a–i^ Means within the same row with different superscript letters differ significantly ( p<0.05 ).

According to Table 5, changes in energy (NEFA, BHBA), protein (urea, albumin, globulin), and lipid (total cholesterol) metabolism parameters were statistically significant in parallel with the progression of gestation and the increase in BCS ( p<0.001 ). Mean serum NEFA levels reached their minimum in the early-pregnancy BCS > 3 group (0.16 mmol L^−1^), while the maximum value was recorded in the dry-period BCS > 3.75 group (0.52 mmol L^−1^) ( p<0.001 ). Specifically, the lowest albumin and globulin levels were found in the dry-period BCS < 3.25 group (30.98 and 29.11 g L^−1^, respectively), while the highest levels were detected in the early-pregnancy BCS > 3 group (37.03 and 34.66 g L^−1^, respectively) ( p<0.05 ). Mean serum urea and total cholesterol levels were lowest in the BCS < 2.5 group during early pregnancy (9.20 and 83.4 mg dL^−1^, respectively), while the highest values were observed in the BCS > 3.75 group during the dry period of pregnancy (16.35 and 113.0 mg dL^−1^, respectively) ( p<0.001 ; Table 5).

**Table 5:** Changes in mean serum NEFA, BHBA, urea, albumin, and total cholesterol levels in Brown Swiss cows across different gestation stages and BCS groups (Mean ± SEM).

Parameters	Early pregnancy	Early pregnancy	Early pregnancy	Mid-pregnancy	Mid-pregnancy	Mid-pregnancy	Dry period	Dry period	Dry period	SEM	p value
	( <2.5 )	(2.5–3.0)	( >3 )	( <2.75 )	(2.75–3.25)	( >3.25 )	( <3.25 )	(3.25–3.75)	( >3.75 )		
NEFA (mmol L^−1^)	0.21^e^ ± 0.01	0.19^f^ ± 0.01	0.16^g^ ± 0.01	0.27^d^ ± 0.01	0.31^c^ ± 0.01	0.36^b^ ± 0.01	0.43^a^ ± 0.01	0.47^a^ ± 0.01	0.52^a^ ± 0.01	0.01	<.001
BHBA (mmol L^−1^)	0.18^c^ ± 0.01	0.23^b^ ± 0.01	0.27^a^ ± 0.01	0.21^b^ ± 0.01	0.21^b^ ± 0.01	0.21^b^ ± 0.01	0.21^b^ ± 0.01	0.21^b^ ± 0.01	0.21^b^ ± 0.01	0.01	0.042
Urea (mg dL^−1^)	9.20^d^ ± 0.16	9.32^d^ ± 0.16	9.61^d^ ± 0.16	10.10^c^ ± 0.16	10.34^c^ ± 0.16	10.55^c^ ± 0.16	11.23^c^ ± 0.16	14.15^b^ ± 0.16	16.35^b^ ± 0.16	0.16	<.001
Albumin (g L^−1^)	35.33^b^ ± 0.28	36.46^a,b^ ± 0.28	37.03^a^ ± 0.28	35.07^b^ ± 0.28	35.68^b^ ± 0.28	36.46^a,b^ ± 0.28	30.98^e^ ± 0.28	31.84^d,e^ ± 0.28	33.07^d^ ± 0.28	0.28	0.012
Globulin (g L^−1^)	30.91^c^ ± 0.31	32.44^c^ ± 0.31	34.66^a^ ± 0.31	31.51^c^ ± 0.31	34.00^b^ ± 0.31	34.20^b^ ± 0.31	29.11^d^ ± 0.31	32.41^c^ ± 0.31	33.72^b^ ± 0.31	0.31	0.035
Total cholesterol (mg dL^−1^)	83.4^g^ ± 0.24	87.1^f,g^ ± 0.24	91.8^f^ ± 0.24	89.2^f,g^ ± 0.24	90.6^f,g^ ± 0.24	97.4^e^ ± 0.24	102.0^e^ ± 0.24	105.1^e^ ± 0.24	113.0^d,e^ ± 0.24	0.24	<.001

^*^ NEFA: non-esterified fatty acid; BHBA: beta-hydroxybutyric acid; EGP: early gestation period; MGP: mid-gestation period; dry: late gestation/dry period; BCS: body condition score; SEM: standard error of the mean. Data were analyzed independently for each gestation stage and BCS group. Statistical significance was set at p<0.05 . ^a–i^ Means within the same row with different superscript letters differ significantly ( p<0.05 ).

## 4 Discussion

### 4.1 Spexin, irisin, visfatin, GRP78, and selected blood parameters

The periparturient period in dairy cattle is characterized by negative energy balance (NEB), intense lipolysis, and systemic cellular stress (McFadden, 2020). Spexin (SPX), reported as a biomarker for metabolic regulation during this phase, is a novel adipokine with pleiotropic functions, including anorexigenic effects (Jeong et al., 2022), body weight control (Walewski et al., 2014), stabilization of insulin secretion (Chen et al., 2021), and energy metabolism regulation (Mikuła et al., 2021; Tran et al., 2022; Sun et al., 2023). In the current study, serum spexin levels in Brown Swiss cattle exhibited a numerical decrease in groups with higher BCS while reaching their highest levels during late lactation and the dry period ( p<0.001 ). Our findings, indicating an increase in mean serum spexin levels with the progression of lactation and pregnancy, are consistent with several reports (Mikuła et al., 2021; Yaprakci and Akkuş, 2025), though they differ from others (Dajnowska et al., 2023). We suggest that this trend reflects a strong inverse relationship between BCS and spexin levels, representing an adaptive response to maintain energy homeostasis, particularly in low-condition animals.

Irisin, a myokine primarily secreted by skeletal muscle, is a pleiotropic molecule that plays a critical role in metabolic adaptation during the periparturient period. It promotes energy expenditure by inducing the browning of white adipose tissue, regulates glucose homeostasis, and reduces insulin resistance (Boström et al., 2012; Zheng et al., 2022). In the current study, serum irisin levels in Brown Swiss cattle increased in parallel with higher BCS ( p<0.001 ). Furthermore, irisin exhibited a gradual increase from the onset of lactation, reaching its peak during the late stage of pregnancy (dry period). These findings are consistent with literature reporting increased serum irisin levels as lactation and pregnancy progress (Çolak et al., 2019; Kızıl et al., 2023). However, our results differ in direction from some studies reporting decreased irisin levels during negative energy balance (NEB) and subclinical ketosis (Eğritağ et al., 2022), although they share a conceptual similarity regarding irisin's role as a metabolic indicator. This difference may be attributed to the positive association between BCS and irisin, as well as the metabolic homeostasis achieved as NEB diminishes with the progression of lactation. Additionally, this trend can be explained by the differentiation of physiological adaptation in healthy Brown Swiss cattle from pathological processes and the potential impact of the breed-specific robust muscular structure on myokine (irisin) secretion.

Visfatin (also known as nicotinamide phosphoribosyltransferase, NAMPT), secreted by adipocytes and immune cells, is a key regulator of energy homeostasis during the periparturient period, characterized by its insulin-mimetic effects, enhancement of glucose utilization, and pro-inflammatory properties (Reverchon et al., 2013). In our study, serum visfatin levels showed a significant increase as lactation progressed from the early to the late stage, peaking in cows with higher body condition scores (Table 2). This increasing trend suggests that visfatin may play a regulatory role in long-term metabolic adaptation and energy balance during advanced lactation stages. These findings are consistent with research reporting increased visfatin levels throughout the lactation and pregnancy processes (Kızıl et al., 2023). Furthermore, they present a conceptual alignment with data indicating decreased visfatin levels in animals experiencing negative energy balance (NEB) (De Koster et al., 2017). This trend can be attributed to the direct relationship between visfatin and adipose tissue mass (BCS), as well as the increased requirement for insulin sensitivity as NEB alleviates in the later stages of lactation. Additionally, this increase in healthy Brown Swiss cattle is suggested to be an adaptive response developed to manage metabolic stress and mobilize energy reserves effectively during the periparturient period.

GRP78, a key biomarker of endoplasmic reticulum (ER) stress, serves as an essential indicator of metabolic demand and cellular adaptation in high-yielding dairy cattle (Ruan et al., 2026). In the current study, it was observed that GRP78 levels were highest during early lactation and the dry period and significantly decreased ( p<0.001 ) as lactation progressed toward the later stages. These results are consistent with literature reporting an elevation of ER stress markers during the periparturient period (Luo et al., 2022). In the present study, GRP78 levels showed different trends depending on the physiological state. While GRP78 levels significantly decreased as lactation progressed from early to late stages (Table 2), a marked increase was observed during the dry period of pregnancy, reaching its peak at 0.46 ng mL^−1^ (Table 4). This decrease during lactation suggests a gradual adaptation of the endoplasmic reticulum to milk synthesis over time. Conversely, the surge during the dry period may reflect the intensive metabolic preparation and cellular stress associated with advanced fetal growth and the upcoming onset of a new lactation cycle. This elevation can be attributed to the physiological stress induced at the cellular level by increased protein synthesis and metabolic workload associated with advanced pregnancy and lactation, which triggers GRP78 release as part of the unfolded protein response (UPR). The rise observed in healthy Brown Swiss cattle is considered a protective mechanism aimed at maintaining cellular integrity and meeting metabolic demands. Therefore, this trend represents a physiological adaptation process rather than pathological ER stress, reflecting the animal's ability to maintain homeostasis under high production pressure.

Serum NEFA and BHBA levels, as primary indicators of negative energy balance (NEB) and lipid mobilization, are decisive parameters for evaluating metabolic adaptation during the periparturient period (Adewuyi et al., 2005). In the current study, mean serum NEFA (0.34–0.41 mmol L^−1^) and BHBA (0.38–0.48 mmol L^−1^) levels at the onset of lactation indicate an absence of severe negative energy balance, remaining below or near the established physiological thresholds ( < 0.40 and <1.0 mmol L^−1^, respectively).. These results indicate that the Brown Swiss cattle in this study effectively managed periparturient metabolic stress, maintained lipid mobilization at a controlled level, and remained within safe limits regarding the risk of ketosis. This resilience is attributed to the metabolic characteristics of the Brown Swiss breed, which tend to be more robust against NEB compared to high-yielding breeds such as Holstein. Brown Swiss cattle appear to mobilize body reserves more conservatively to compensate for energy deficits. Furthermore, in contrast to pathological conditions where severe NEB leads to a decrease in irisin levels (Eğritağ et al., 2022), the increasing trend in adipokines (irisin, spexin, and visfatin) observed in our study reflects a successful physiological adaptation process in healthy animals. This suggests that the coordination between adipose-derived hormones and cellular stress responses (GRP78) effectively supports metabolic homeostasis in this breed.

Serum albumin levels (reflecting the synthetic capacity of the liver and overall protein status), globulin levels (as indicators of immune response), and urea levels (the end product of nitrogen metabolism) provide essential information regarding the protein–energy balance of the diet and general health status (Osorio et al., 2014). In the current study, serum albumin (30.98–38.08 g L^−1^) and globulin (29.11–36.36 g L^−1^) concentrations maintained a narrow range throughout the periparturient period. In our study, serum urea levels were observed to decrease toward the late lactation period, coinciding with a significant decline in NEFA concentrations. This reduction indicates a shift toward metabolic stability, improved energy balance, and more efficient utilization of dietary protein as the animals transition out of the high-stress early lactation phase. This suggests an improvement in energy balance and the stabilization of protein metabolism. While elevated NEFA in early lactation reflects intense lipid mobilization (negative energy balance), the subsequent rise in urea within physiological limits indicates a shift toward metabolic stability and efficient utilization of dietary protein as the animals transition out of the high-stress production phase. This homeostatic stability suggests that the hepatic synthetic capacity of the animals was preserved and that the immune system remained resilient against metabolic stress. This resilience can be attributed to the adequacy of the dietary protein content in meeting requirements during the periparturient period and the ability of Brown Swiss cattle to maintain protein synthesis capacity under lactation stress. Furthermore, the periodic fluctuations observed in urea levels are considered a physiological response to changes in the dietary protein-to-energy ratio or the rate of tissue protein mobilization.

Serum total cholesterol levels in cattle are directly associated with lipoprotein synthesis and energy intake, typically exhibiting an upward trend as lactation progresses postpartum (Kessler et al., 2014). The increase in total cholesterol observed in the current study (22 % during gestation and 34 % during lactation) is in full agreement with previous reports (Cozzi et al., 2011; Kessler et al., 2014). In cattle, serum cholesterol concentrations rise due to the heightened energy demand following the onset of lactation and the increased requirement for lipid precursors (lipoproteins) necessary for milk synthesis (Van Den Top et al., 1995). The literature indicates that cholesterol levels – which remain low during early lactation, the period when negative energy balance (NEB) is most severe – gradually increase in later stages as feed intake improves, and energy balance stabilizes (Kessler et al., 2014). Indeed, the rise in cholesterol levels from 114 to 154 mg dL^−1^ in parallel with the progression of lactation in our study confirms that the animals successfully transitioned out of NEB and that the liver's lipoprotein synthesis capacity was enhanced. This trend can be attributed to the stimulation of hepatic synthesis mechanisms by the increasing cholesterol demand for milk production and the rise in exogenous lipid intake associated with higher dry matter intake as lactation advances. Furthermore, it is suggested that the regulatory effects of adipokines, such as irisin and spexin, on cholesterol metabolism also play a significant role in shaping this metabolic profile.

### 4.2 Limitations

Although this study provides comprehensive multivariate insights into metabolic, adipokine, and cellular stress dynamics in multiparous Brown Swiss cows, certain methodological limitations should be acknowledged. Firstly, while commercial ELISA kits were utilized to quantify circulating concentrations of novel adipokines and myokines (iris, spexin, visfatin) and ER stress markers (GRP78), the analytical specificity and cross-reactivity of commercially available ELISA assays for bovine matrices remain a topic of ongoing discussion in the literature – particularly for cleaved peptides such as irisin. Although internal assay validation protocols (e.g., intra- and inter-assay coefficients of variation) were strictly maintained within acceptable thresholds in the present study, future investigations employing mass spectrometry (e.g., LC-MS/MS) or western blotting are recommended to further validate epitope specificity and absolute peptide quantification in bovine serum. Secondly, while our dataset spans a robust sample size across multiple physiological stages and body condition score categories, functional gene expression assays in target tissues (e.g., adipose or hepatic tissue biopsies) were not performed, which would complement the circulating biomarker profile.

## 5 Conclusions

In conclusion, this study demonstrates a highly significant interaction ( p<0.001 ) between conventional metabolic profile parameters (NEFA, BHBA), novel adipokines (spexin, irisin, visfatin), and the cellular stress response marker (GRP78) in periparturient Brown Swiss cattle. The findings reveal that as lactation progresses, and BCS fluctuates, mean serum spexin levels exhibit an antagonistic relationship with lipolysis intensity (NEFA), while irisin levels show a synchronized change with indicators of metabolic stability. Notably, our data identify the dry period as the most critical physiological phase, characterized by peak ER stress and lipid mobilization. These patterns suggest that spexin and GRP78 can be utilized as potent prognostic biomarkers for the early detection of metabolic stress and ketosis risk. Furthermore, the elevated irisin levels observed may reflect the unique metabolic resilience associated with the characteristic muscular conformation of the Brown Swiss breed. Therefore, rather than employing general herd management, targeted nutritional interventions and specific antioxidant strategies are essential during the dry period to mitigate cellular stress and ensure a successful transition to the next lactation. These results provide a robust framework for developing more effective, breed-specific monitoring protocols aimed at enhancing animal welfare and productivity in herd health management.

## Data Availability

The datasets generated and analyzed during the current study are not publicly available due to institutional research data protection protocols and privacy agreements associated with commercial dairy farm operations but are available from the corresponding author upon reasonable request.
